# Musculoskeletal practices for the preparticipation physical examination

**DOI:** 10.1186/s13102-021-00316-x

**Published:** 2021-08-04

**Authors:** Connor Corrente, Matthew Silvis, Joseph Murphy, Robert Gallo, Cayce Onks

**Affiliations:** 1 Penn State College of Medicine, 700 HMC Crescent Road, Hershey, PA 17033 USA; 2 Department of Orthopedics and Rehabilitation & Family and Community Medicine, Penn State Milton S. Hershey Medical Center, 500 University Drive, H154, Hershey, PA 17033 USA; 3 Department of Athletic Training, Lebanon Valley College, 212 Arnold Pavilion, Annville, PA 17003 USA; 4 Department of Orthopedics and Rehabilitation, Penn State Milton S. Hershey Medical Center, 500 University Drive, Hershey, PA 17033 USA

**Keywords:** Musculoskeletal (MSK), Preparticipation Physical Examination (PPE), Injury prevention, MSK screening exam

## Abstract

**Background:**

Little is known about the musculoskeletal (MSK) exam providers use during the Preparticipation Physical Examination (PPE). The primary aims of this study were to determine current practice with regards to the MSK screening exam, if goals are being met, and if there may be opportunities for improvement.

**Methods:**

This cross-sectional survey-based study utilized a REDCap instrument that was distributed to members of the American Academy of Family Physicians (AAFP) and the American Medical Society for Sports Medicine (AMSSM). Questions focused on participant demographics and MSK exam practices for the PPE. Descriptive statistics were used.

**Results:**

The study had a total of 616 participants with a response rate of 9 %. The majority of respondents (82 %) were familiar with the 4th Edition PPE Monograph and 80 % either moderately or strongly agreed that they use this as a guideline for their MSK screening exam. The 90 s MSK screening test was implemented by 52 % of the participants. The majority of participants use an orthopedic exam as part of their PPE (82 %). Ninety-two percent of participants felt satisfied that their MSK exam would screen for current injury, while only 42 % were satisfied that it effectively screened for future injury. 86 % of participants agree that the MSK exam should be performed, while 26 % said that they don’t perform a physical exam at all.

**Discussion:**

There is a lack of understanding of the PPE Monograph as there is wide variability in MSK screening techniques providers use despite the majority of participants being familiar with the guidelines described in the monograph. Additionally, providers don’t believe that the MSK exam screens for future injury.

**Conclusions:**

The goals of the MSK portion of the 4th Edition PPE monograph are not adequately being met and there is a need for further research to validate screening exams for the prevention of MSK injury.

## Background

Athletes aged 5–14 suffered an estimated 5.6 million recreation related injuries requiring medical attention from 2011 to 2014 [1] The High School Sports-Related Injury Surveillance Study showed that ankle and knee injuries were two of the most common high school sports-related injuries, accounting for over 30 % of all high school sports-related injuries in the 2018-19 school year [2]. Longer-term consequences of sports injuries include predisposition to recurring injuries, earlier sports termination/dropout, and potentially compromised physical and psychological health [3].

The current standard of care for screening athletes at risk for injury and illness is the Preparticipation Physical Examination (PPE), but there has been no conclusive evidence that supports the effectiveness of the musculoskeletal (MSK) PPE to accurately identify or prevent at risk populations from injury [4, 5]. Despite not having a validated screening exam, the musculoskeletal portion of the PPE has been found to be the most common cause of disqualification for athletes, mostly through the history and review of systems form [6].

The primary goal of the PPE as described in the 4th Edition PPE Monograph, which is now an older version but was used at the time of this study, is to (1) screen for conditions that may be life-threatening or disabling, and (2) screen for conditions that may predispose individuals to injury or illness [5]. Currently, there is no standardization for healthcare providers on how the PPE is completed, and there are questions surrounding the effectiveness of the PPE in meeting its objectives to properly screen athletes. In a study completed in 2014, it was found that healthcare providers overall were unaware of the PPE screening guidelines, and that knowledge of the Fourth Edition PPE Monograph led to increased satisfaction with the PPE as a screening tool [7].

Effective screening tests must satisfy 2 requirements as described by the US Preventive Services Task Force: (1) the test must be able to detect abnormalities earlier than without screening and (2) the screening must be accurate [8]. Due to the need to understand more completely how to better screen athletes for MSK injury we designed a survey to gain information from physicians performing PPE’s. Our aims were to understand current physician practices in regard to the MSK screening exam, if physicians who perform PPEs feel the current goals of the PPE are being met, and if there may be opportunities for education in teaching of the MSK screening examination.

## Methods

 Following institutional review board approval, members from the American Academy of Family Physicians (AAFP) and the American Medical Society for Sports Medicine (AMSSM) were recruited for the study. Inclusion criteria included current practicing physicians in primary care, primary care sports medicine, and sports orthopedics that perform the PPE as part of his/her practice. Exclusion criteria included providers who are not currently practicing, and individuals that do not perform the PPE as part of their practice.

The study utilized a cross-sectional survey-based REDCap instrument for data collection. 3,000 members of the AAFP were mailed postcards that contained a link and scannable QR code for participants to access the survey and 3,871 members of the AMSSM were sent an email that was generated through the organization on two occasions, one month apart, containing a link to the survey.

The survey contained a total of 24 questions and was separated into two main sections: a series of questions regarding the demographics of each participant followed by questions pertaining to participants’ MSK screening practices for the PPE. Demographic questions investigated the number of years of clinical practice, specialty of provider, location of practice, and level of athlete (middle school through professional) typically seen. The MSK screening questions involved a variety of Likert scale, multiple select, and yes/no questions exploring providers’ satisfaction that their screen is appropriate for current or future injury, knowledge of the 4th Edition PPE Monograph, what physical exam tests are commonly performed in the PPE, thoughts on the value of the MSK screening exam, and if the PPE is adjusted based on athlete level. Additionally, information regarding the number of PPEs performed per year, most common age group of athletes screened, and facilities in which the PPEs are being performed in was collected. Prior to administration, the survey was reviewed for clarity by 3 board certified sports medicine physicians (two primary care and one orthopedic), 2 family practice physicians, and 1 certified athletic trainer who were not involved in the creation of the questionnaire. Descriptive statistics were used in the analysis of the data, comparing frequency and percentages of responses for each answer choice.

## Results

A total of 616 out of 6871 participants (9 %) responded to the survey. The majority of participants, 72 %, were family medicine physicians with the remaining 28 % being composed of orthopedic, internal medicine, emergency medicine, pediatric, and physical medicine and rehabilitation physicians. Participants practiced across 46 states collectively.

**Fig. 1:**
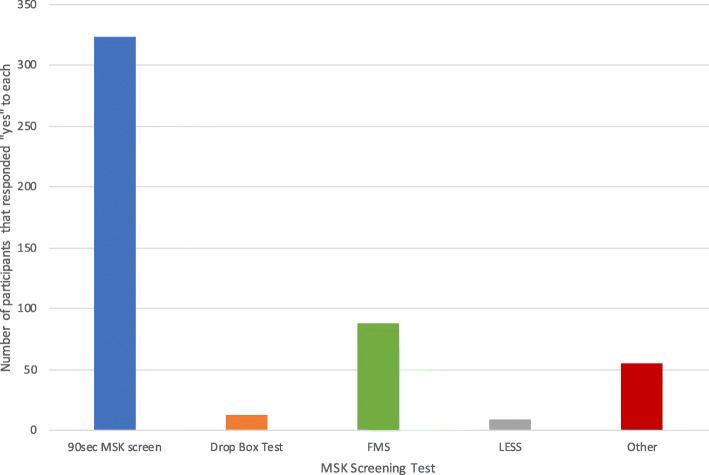
Variability in MSK screening tests used during the PPE (*n* = 616). The 90 second MSK screening exam was the most common among all participants. (*FMS* = Functional Movement Screen; *LESS* = Landing Error Scoring System; *Other* = mixed responses including Duck Walk, individual joint examinations, and focused examination of areas based on history)

Variability in the MSK screening exam is depicted in Fig. 1. The 90 s MSK screening test was implemented by 52 % of the participants, the most common among physicians. The majority of participants who perform a MSK exam use an orthopedic exam as part of their PPE (82 %). Twenty-six percent of participants reported that they do not perform a physical exam at all as part of their MSK screening examination, while 86 % of participants agreed that the MSK exam should be performed. Ninety-two percent of participants felt satisfied that their MSK examination screened for current injury, while only 42 % were satisfied that it effectively screened for future injury. One of the main barriers to performing portions of the MSK screen was time (46 %), while 22 % reported lack of evidence for the exam as their reason.

Educationally, 51 % received training for the MSK PPE in residency, and 62 % had training in fellowship. The majority of respondents (82 %) were familiar with the 4th Edition PPE Monograph and 80 % either moderately or strongly agreed that they use this as a guideline for their MSK screening exam. Data comparing knowledge of the monograph and number of PPEs completed yearly showed that of the participants that complete more than 100 PPEs yearly, there is a higher percent of participants (88 %) with knowledge of the 4th Edition PPE Monograph compared to those who do not have knowledge of the PPE monograph (12 %). Additionally, of those that were aware of the 4th Edition PPE Monograph, most participants (59 %) completed more than 75 PPEs per year, while those that completed 25 or less PPEs per year only made up 11 %. 13 % reported no knowledge of the 90 s MSK screening test that is included in the 4th Edition PPE Monograph. These results are summarized in Table 1.

**Table 1:** Participant Demographics and PPE Experience Data

Total providers contacted	6871
Total responses	616^a^
Practice location (*n* = 496)	
Rural	56 (11.29%)
Suburban	263 (53.02%)
Urban	177 (35.69%)
Years practicing (*n* = 497)	
1-5	184 (37.02%)
6-10	96 (19.32%)
11-15	65 (13.08%)
15-20	54 (10.87%)
>20	98 (19.72%)
Primary Practice Setting (*n* = 616)	
Outpatient Primary Care	147 (23.86%)
General Orthopedics	17 (2.76%)
Sports Orthopedics	78 (12.66%)
Primary Care Sports Medicine	314 (50.97%)
Pediatrics	8 (1.30%)
Other	36 (5.84%)
Residency Completed (*n* = 504)	
Family Medicine	360 (71.43%)
Internal Medicine	30 (5.95%)
Orthopedic Surgery	1 (0.20%)
Emergency Medicine	18 (3.57%)
Pediatrics	47 (9.33%)
Physical Medicine and Rehabilitation	35 (6.94%)
Other	13 (2.58%)
Sports Medicine Fellowship Training (*n* = 496)	
Yes	439 (88.51%)
No	57 (11.49%)
Team Physician (*n* = 495)	
Yes	393 (79.39%)
No	102 (20.61%)
Level of Sport Covered (*n* = 616)	
High School	283 (45.94%)
Club	75 (12.18%)
College	273 (44.32%)
Professional	122 (19.81%)
National Team	51 (8.28%)
Other (Dance, military, semi-pro, roller derby, endurance events)	12 (1.95%)
Training for PPE (*n* = 616)	
Medical School	139 (22.56%)
Residency	314 (50.97%)
Fellowship	380 (61.69%)
Continuing Medical Education	123 (19.97%)
PE Literature	174 (28.25%)
Other (undergraduate education, experience, colleagues)	9 (1.46%)
Familiarization of 4^th^ Edition PPE Monograph (*n* = 466)	
Yes	383 (82.19%)
No	83 (17.81%)
Use of PPE Monograph as guideline (*n* = 382)	
Disagree Strongly	4 (1.05%)
Disagree Moderately	8 (2.09%)
Disagree Slightly	13 (3.40%)
Agree Slightly	50 (13.09%)
Agree Moderately	180 (47.12%)
Agree Strongly	127 (33.25%)
Satisfied with MSK screening exam for CURRENT injury (*n* = 466)	
Yes	431 (92.49%)
No	35 (7.51%)
Satisfied with MSK screening exam for FUTURE injury (*n* = 466)	
Yes	194 (41.63%)
No	272 (58.37%)
Location where PPE is performed most commonly (*n* = 616)	
Normal office patient encounter	370 (60.06%)
On-field with team	16 (2.60%)
Gymnasium	201 (32.63%)
Physical Therapy Clinic	18 (2.92%)
Athletic Training Room	264 (42.86%)
Other	43 (6.98%)
Age groups most commonly assessed (years) (*n* = 466)	
<12	5 (1.07%)
13-18	258 (55.36%)
18-25	198 (42.49%)
>25	5 (1.07%)
Number of PPE performed yearly (*n* = 466)	
0-25	63 (13.52%)
26-50	77 (16.52%)
51-75	68 (14.59%)
76-100	54 (11.59%)
>100	204 (43.78%)

Details the demographic data of respondents in the study as well as prior experiences and opinions in regards to the PPE

^a^Total responses may not equal total number of participants due to missing data

## Discussion

There are a variety of approaches that are used for the MSK screening examination during the PPE. Most participants believe that the MSK exam should be performed and that it effectively screens for current injuries, but the majority of providers do not believe that the MSK exam adequately screens for future injury. This data confirmed our perception that providers do not believe in the predictive ability of the screen. To that end almost a third of respondents reported that they do not perform a physical exam at all. This perception is also in agreement with literature investigating the effectiveness of the MSK PPE, which shows there has been no convincing evidence that the MSK PPE is effective at accurately identifying or preventing at risk athletes from injury [4, 9, 10].

Most participants were aware of the 4th Edition PPE Monograph and reported that they primarily use it as their guideline. Even with participants reporting using the monograph as their guideline for the MSK screen, there is still a lot of variability in how the screen is being performed, with half of the participants using the 90 s MSK screening test, 14 % using the Functional Movement Screen (FMS), and close to a third not using a physical exam at all for the screen. Approximately 46 % of participants reported lack of time as a barrier to performing some portions of the MSK PPE, providing some explanation as to why this variability may exist. Another explanation for the inconsistency could be due to educational differences amongst providers as there was variability in where providers were taught the MSK exam. Fifty-one percent of respondents received training for the MSK PPE in residency and 62 % in fellowship programs, which highlights the lack of standardization in curriculums. As curriculums are updated there should be discussions regarding the value of the MSK screening and if standardization can improve the quality of MSK screenings provided.

Our findings support a lack of understanding of the PPE monograph given the wide variability in MSK screening exam techniques used while being aware of the PPE guidelines. This calls attention to the need for continued standardization of the MSK screening exam, as well as further research to validate objective screening exams for the prevention of MSK injury. Screening exams should be explored to look at the relationships between screening tests and risk factors in relevant populations to determine what tests are appropriate and effective in identifying high-risk populations [9]. A recent study by Teyhen et al. was able to show in a military population that the sum of a number of risk factors was able to produce a highly sensitive model for identifying those at risk for MSK injury [11]. This highlights that future MSK screenings should not focus on a single screen, but that a multivariate model with multiple risk factors could successfully identify a high-risk population. Injury prevention programs have shown to be effective in reducing injuries in athletes across a range of sports [12–14]. Identifying the high-risk population would provide an opportunity to direct limited prevention resources to the most at-risk individuals with the ultimate goal to reduce overall injury risk.

There are several limitations to our study. Our survey was the first that we are aware of that looked to gain provider insight to the MSK exam. As with any questionnaire-based research study, limitations regarding validation of the instrument used is of paramount concern. Validation of a questionnaire requires a process to determine construct, criterion, and content validity, amongst others. We addressed content validity through independent review of the questionnaire by 3 sports medicine physicians (two primary care and one orthopedic), 2 family medicine physicians, and 1 certified athletic trainer not involved in the creation of the questionnaire. Because there are no other instruments available to assess similar information, we were unable to assess criterion validity. Given the simple design of the questionnaire, we did not believe that it was necessary to assess construct validity [15]. The study was also limited due to a low response rate of 9 %, however, other web-based survey studies targeting members of the AMSSM had similar response rates [16–18]. Additionally, it is important to note that the response rate is slighter higher than 9 % due to crossover of providers being active members of both the AAFP and AMSSM. The low response rate from providers specializing in fields other than family medicine did not allow for further analysis across specialties.

## Conclusions

This study provides evidence for variability in MSK screening practices. It also highlights physician concerns that the goals of the PPE are not being met with the MSK screening. Finally, there may be a need to improve educational efforts in residencies and fellowships. Future steps include creation of a validated MSK screening model, similar to other primary prevention screening models (CAD, Colon cancer, etc.), that accomplishes the goals of the MSK portion of the PPE, which can then be implemented to be a standard part of the PPE exam.

## Data Availability

The datasets generated and/or analyzed during the current study are available in the REDCap repository and available from the corresponding author on reasonable request.

## Abbreviations

MSK: Musculoskeletal
PPE: Preparticipation Physical Examination
AAFP: American Academy of Family Physicians
AMSSM: American Medical Society for Sports Medicine
FMS: Functional Movement Screen
